# Author Correction: Direct observation of a crescent-shape chromosome in expanded *Bacillus subtilis* cells

**DOI:** 10.1038/s41467-024-47723-5

**Published:** 2024-04-16

**Authors:** Miloš Tišma, Florian Patrick Bock, Jacob Kerssemakers, Hammam Antar, Aleksandre Japaridze, Stephan Gruber, Cees Dekker

**Affiliations:** 1 Department of Bionanoscience, Kavli Institute of Nanoscience Delft, Delft University of Technology, Delft, Netherlands; 2 Department of Fundamental Microbiology (DMF), Faculty of Biology and Medicine (FBM), University of Lausanne (UNIL), Lausanne, Switzerland

**Keywords:** Bacteria, Biophysics

Correction to: *Nature Communication* 10.1038/s41467-024-47094-x, published online 28 March 2024

The original version of this article contained an error in the “Acknowledgement “section.

The original version read “We also acknowledge funding for the work in S.G. lab by the Swiss National Science Foundation (grant number: 310030L_170242).”

This has been amended to “We also acknowledge funding for the work in S.G. lab by the Swiss National Science Foundation (grant number: 310030_197770).”

This has now been corrected in both the PDF and HTML versions of the Article.

