# Supplementary material for: Direct observation of a crescent-shape chromosome in expanded Bacillus subtilis cells
Source: Nat Commun. 2024 Mar 28;15:2737. doi: 10.1038/s41467-024-47094-x (PMC10979009; doi:10.1038/s41467-024-47094-x)
Supplement: Supplementary file 1 — Supplementary Information [file 41467_2024_47094_MOESM1_ESM.pdf]

Supplementary material for

Direct observation of a crescent-shape chromosome in expanded *Bacillus subtilis* cells

Miloš Tišma[1], Florian Patrick Bock[2], Jacob Kerssemakers[1], Hammam Antar[2], Aleksandre Japaridze[1], Stephan Gruber[2], Cees Dekker[1*]

[1] Department of Bionanoscience, Kavli Institute of Nanoscience Delft, Delft University of Technology; Delft, Netherlands.

[2] Department of Fundamental Microbiology (DMF), Faculty of Biology and Medicine (FBM), University of Lausanne (UNIL); Lausanne, Switzerland.

* Corresponding authors: C.Dekker@tudelft.nl

This file includes:

Supplementary figures S1-S18

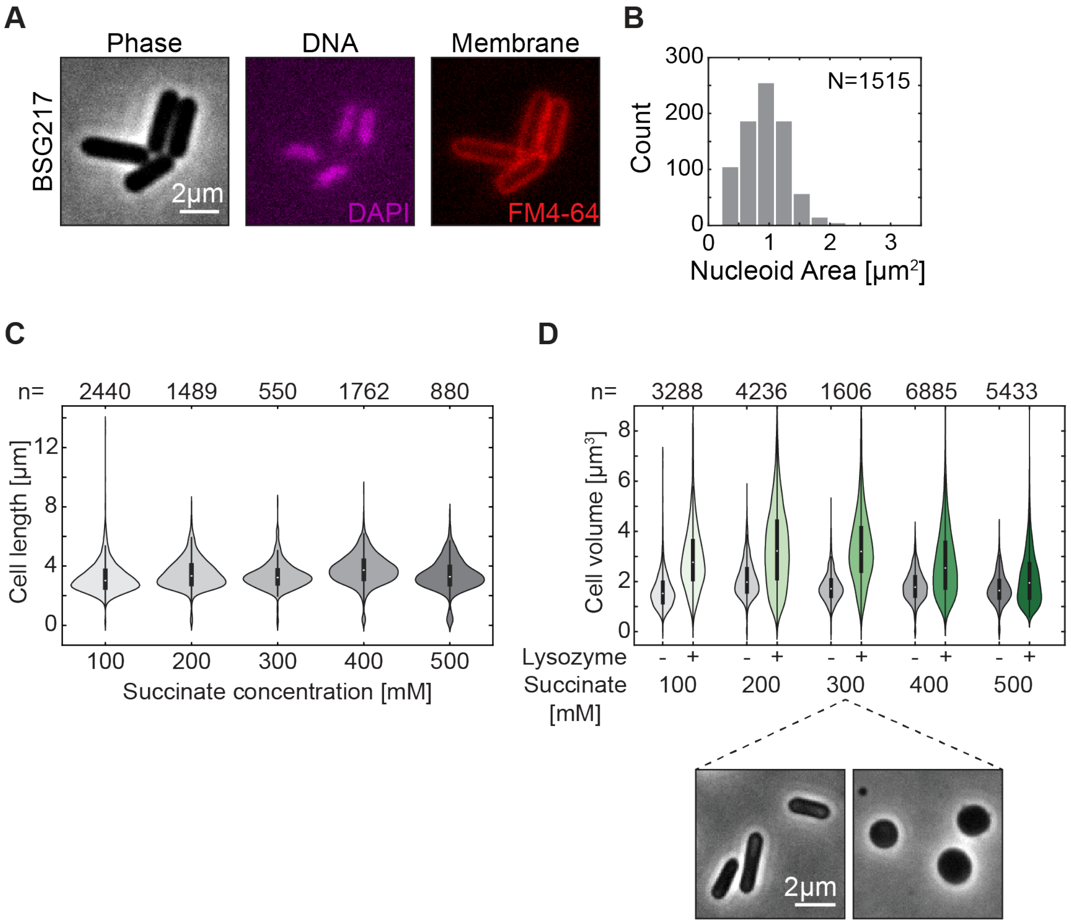

Figure S1. Transformation from cylindrical to spherical cell shape leads to an increase in volume. A) Phase and fluorescent images of the BSG217 strain (see Table S1), with labelled DNA and cell membrane, after replication halt at 37°C for 60 min. **B)** Nucleoid area in replication halted cells shown in A). Mean nucleoid length and width were l=1.28 ± 0.49 µm and w=0.72 ± 0.09 µm (mean ± std, N = 1515). **C)** Longitudinal cell length of rod-shaped *Bacillus subtilis* cells (strain BSG217) grown in SMM+MSM medium of different osmolarities. **D)** Cell volumes of rod-shaped and spherical cells (see Methods and ref 2) grown in the same SMM+MSM medium of different osmolarities. Gray data represent samples that were not exposed to lysozyme treatments; green data represent cells exposed to lysozyme treatment (see Methods).

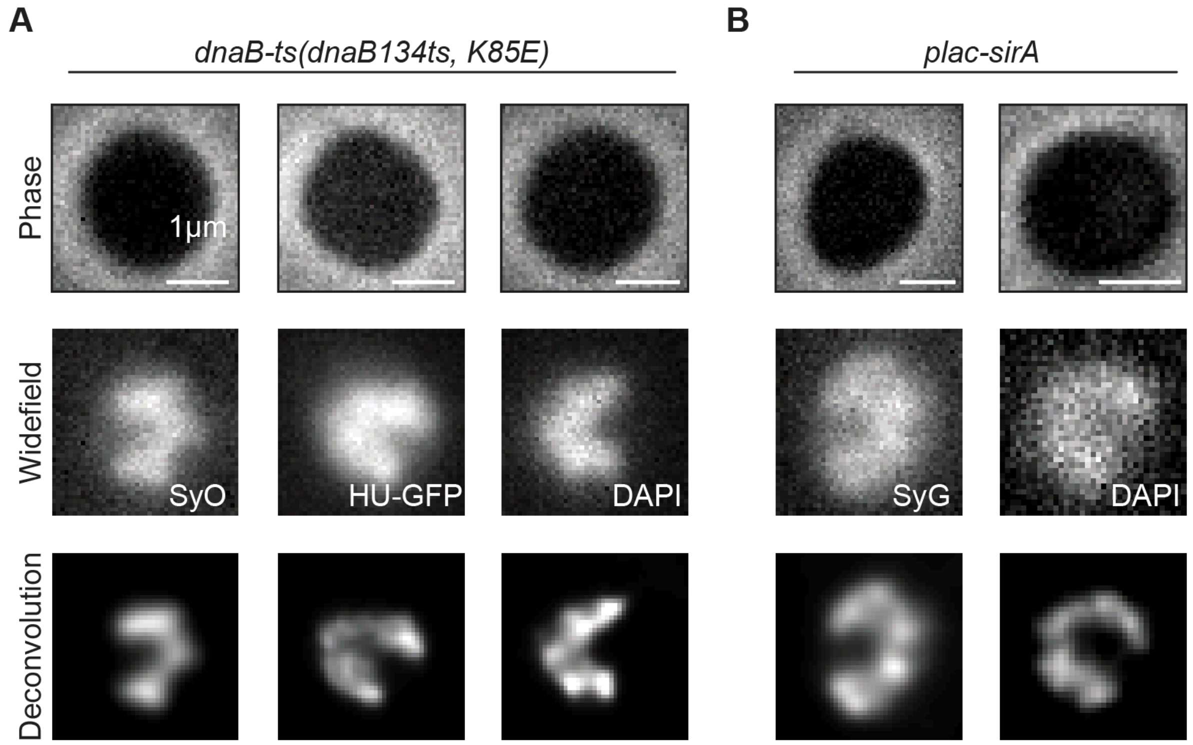

Figure S2. ***Bacillus subtilis*** **chromosome adopts a crescent shape regardless of replication halt strategy or DNA-visualization dye. A)** Phase and fluorescent images of crescent chromosomes in BSG217 cells after lysozyme treatment (400 µg/ml for 20 min) using different DNA dyes (SYTOXOrange (250 µg/ml), GFP-fusion, DAPI (3 µg/ml)). Top to bottom – phase image of spherical bacterial cells, widefield fluorescence image, and the same images after deconvolution via Huygens Professional software (see Methods). **B)** Same as A) for the BSG4595 strain that is replication halted using 2 mM IPTG for 90 min.

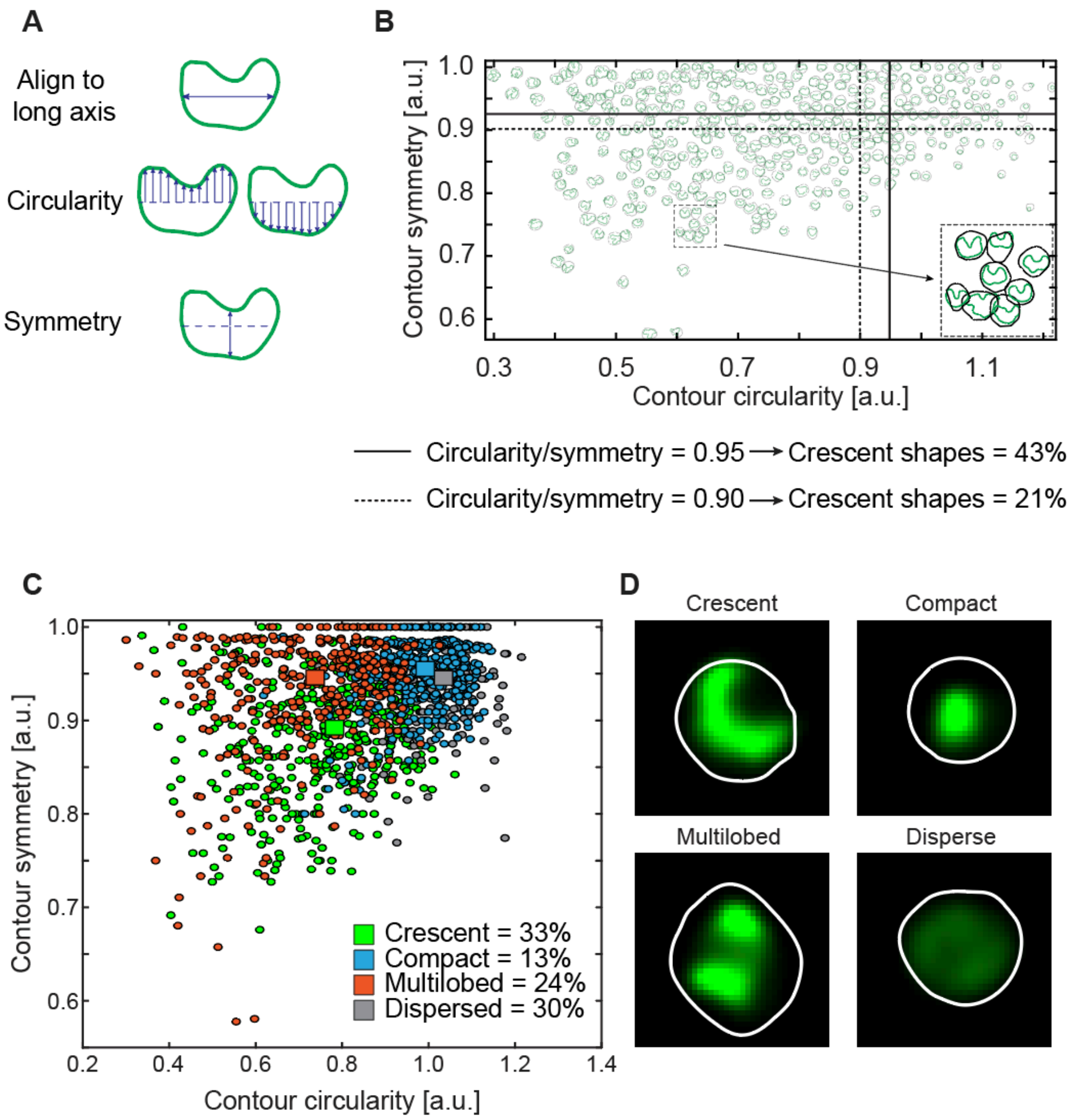

Figure S3. Quantification of crescent-shaped chromosomes in spherical cells that were exposed to hypoosmotic medium. A) Graphical representation of the stepwise selection of crescent chromosomes. Initially selected chromosomes as Oufti objects (see Methods) are positioned along their long axis. Then a 'circularity estimator' is applied that estimates the number of equidistant points in the top and bottom of the mid-axis. Finally, a 'symmetry estimator' measures the relative difference in the distance from the midpoint to the mid-axis. **B)** Cells plotted by their circularity and symmetry. Green line represents the Oufti object outline (which characterizes the chromosome contour)[2]. n = 1321. Selection at threshold of 0.90 (dashed line) or 0.95 (full line) for the ration of circularity/symmetry results in 21% or 43% crescent shapes, respectively. **C)** Blinded manual classification of the chromosome shape

shown in B). The colored squares represent a mean value of symmetry and circularity estimates for the corresponding population. **D)** Fluorescent images of the different shapes obtained in C). White line represents cell outline based on a brightfield image.

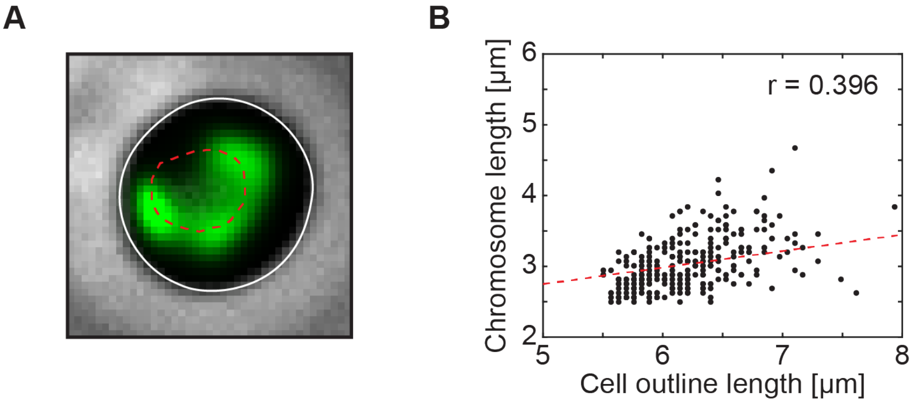

Figure S4. Crescent chromosome size is only weakly correlated with final cell size. A) The micrograph shows the cell outline in white full line, and crescent chromosome contour in red dashed line. **B)** Chromosome contour length versus cell-boundary contour length in spherical cells BSG4595. Black dots represent individual data points. N = 292, r = 0.3965.

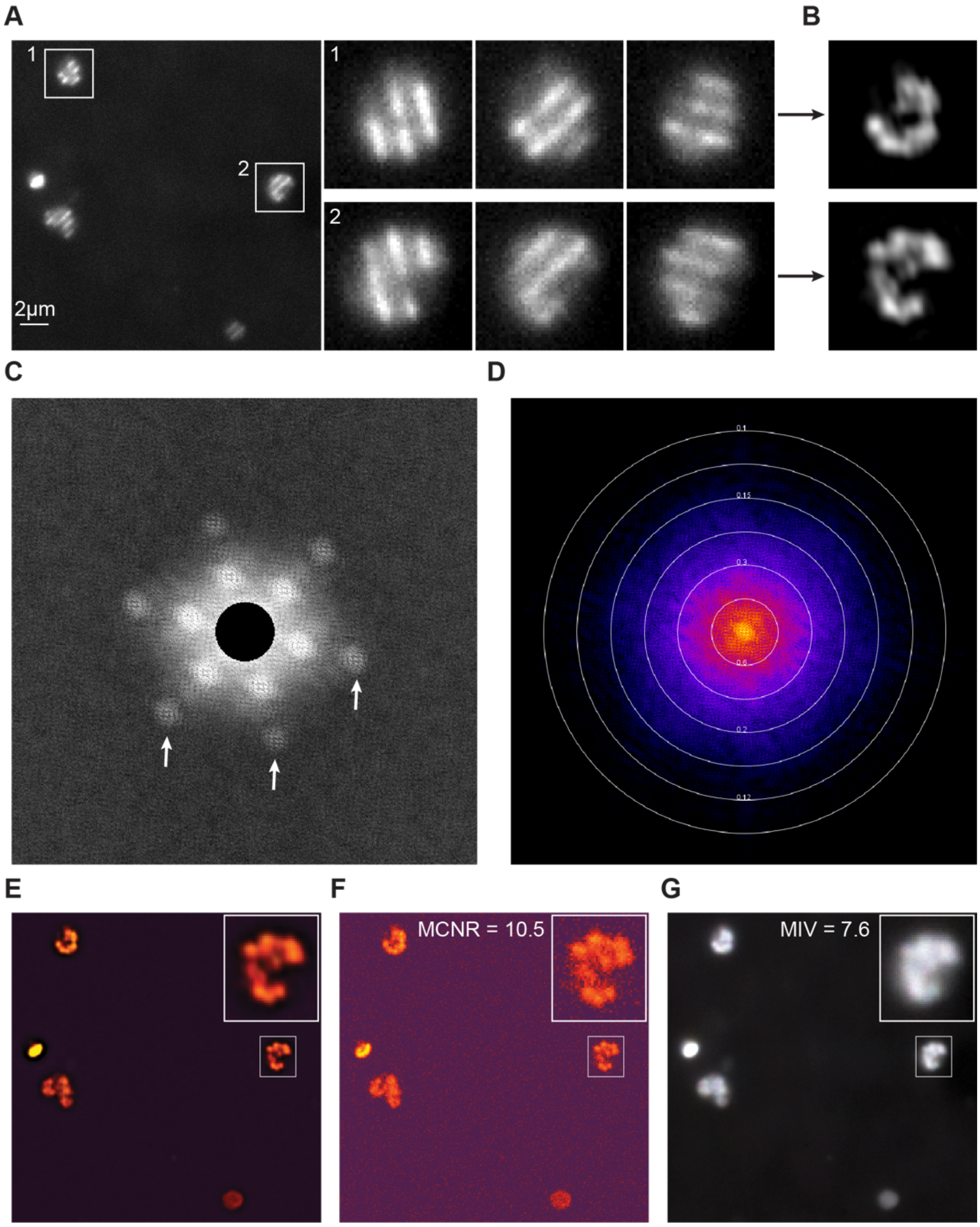

Figure S5. Structured Illumination Microscopy image controls. A) Individual frame from the raw image sequences of 5x3 imaging (position x angle) in the SIM microscope. Two cells of the strain BSG217 (see Table S1) are zoomed and shown with the raw image in the three frames. **B)** Reconstructed SIM image of these individual cells. **C)** Fourier projection of the raw data of the full image (in panel A) in reciprocal space. The image shows the first and second order point of high frequency in all the angles (white arrows), which we used as mandatory to pass the quality control. **D)** Fourier-space image of the reconstructed image overlaid with concentric circles showing corresponding the spatial resolution (in µm). Based on the image, we estimate a resolution of ~0.16 µm. **E)** Modulation Contrast map for reconstructed bottom image from panel A-B). **F)** Same as in E) but for raw image. The MCNR (modulation contrast-

to-noise ratio) passes the SIMCheck quality control[3]. **G)** Motion and illumination variation (MIV) in different angles and frames. The gray image with an absence of colors indicates a high stability and low variation between angles during illumination in the SIM imaging, which ensures that the total imaging sequence for one image is faster than any visible movements of the *B. subtilis* chromosome.

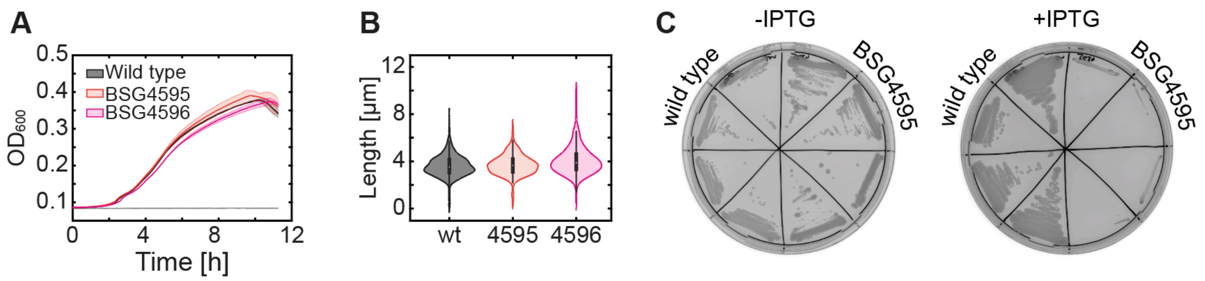

Figure S6. Bacterial growth and phenotype is not affected by genetic edits to *parB* and *amyE* loci. A) Tecan plate reader growth curves (see Methods) for B. subtilis 1A700 strain and BSG4596, BSG4595 strains, carrying P_{lac}*-sirA* and P_{lac}*-sirA* + *parB-mScarlet* (used in extensive quantification in Fig. 2), respectively. **B)** Longitudinal cell length in phase images for the wild-type strain and P_{lac}*-sirA* containing modified strains. **C)** Plating assay showing replication halt in the absence (left) or presence (right) of 2 mM IPTG for the strain BSG4595.

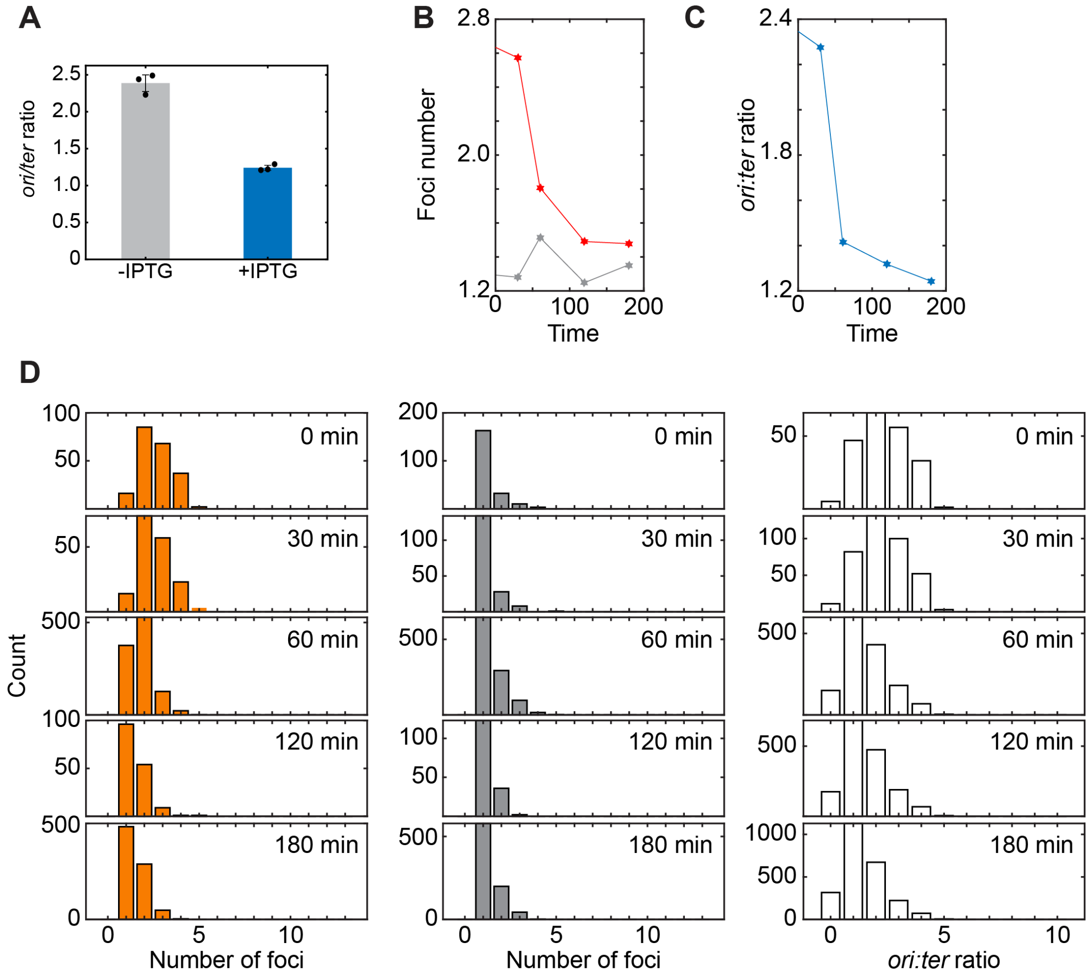

Figure S7. Chromosome replication is efficiently halted upon SirA expression. A) qPCR determination of *ori:ter* ratio in the strain BSG4595. Gray bar shows an untreated sample, and the blue bar shows a sample treated with 2 mM IPTG for 2.5 h. Both conditions were done in biological triplicates with technical triplicates for each of the colonies. Black dots represent an average value of a technical triplicate, for each biological triplicate. Error bars represent a standard deviation from three biological triplicates. **B)** Number of *ori* (red) and *ter* (gray) foci in the strain BSG5522 upon replication halt via IPTG (2mM). **C)** Average ratio of *ori* to *ter* ratio in the same sample. **D)** Time-course measurement of the number of *ori* (orange), *ter* (gray) 0-180 min after the addition of IPTG (2mM). Individual *ori-ter* ratios are presented on the right.

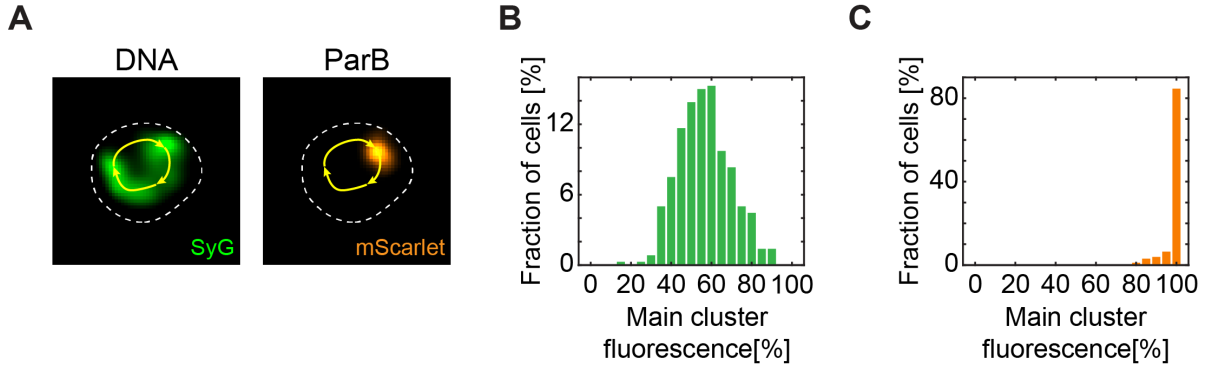

Figure S8. DNA and ParB clusters adopt different fractions of the total signal. A) Representative fluorescence images of the crescent chromosome and ParB focus (cf. Fig. 2) in strain BSG4595. Dashed white line represents cell outline, and yellow arrows represent a sketch of the contour line measurement along the crescent chromosome. **B)** Relative DNA presence (based on fluorescent signal, see Methods) in the main cluster compared to the total DNA signal within the crescent chromosome. **C)** Same for the ParB signal.

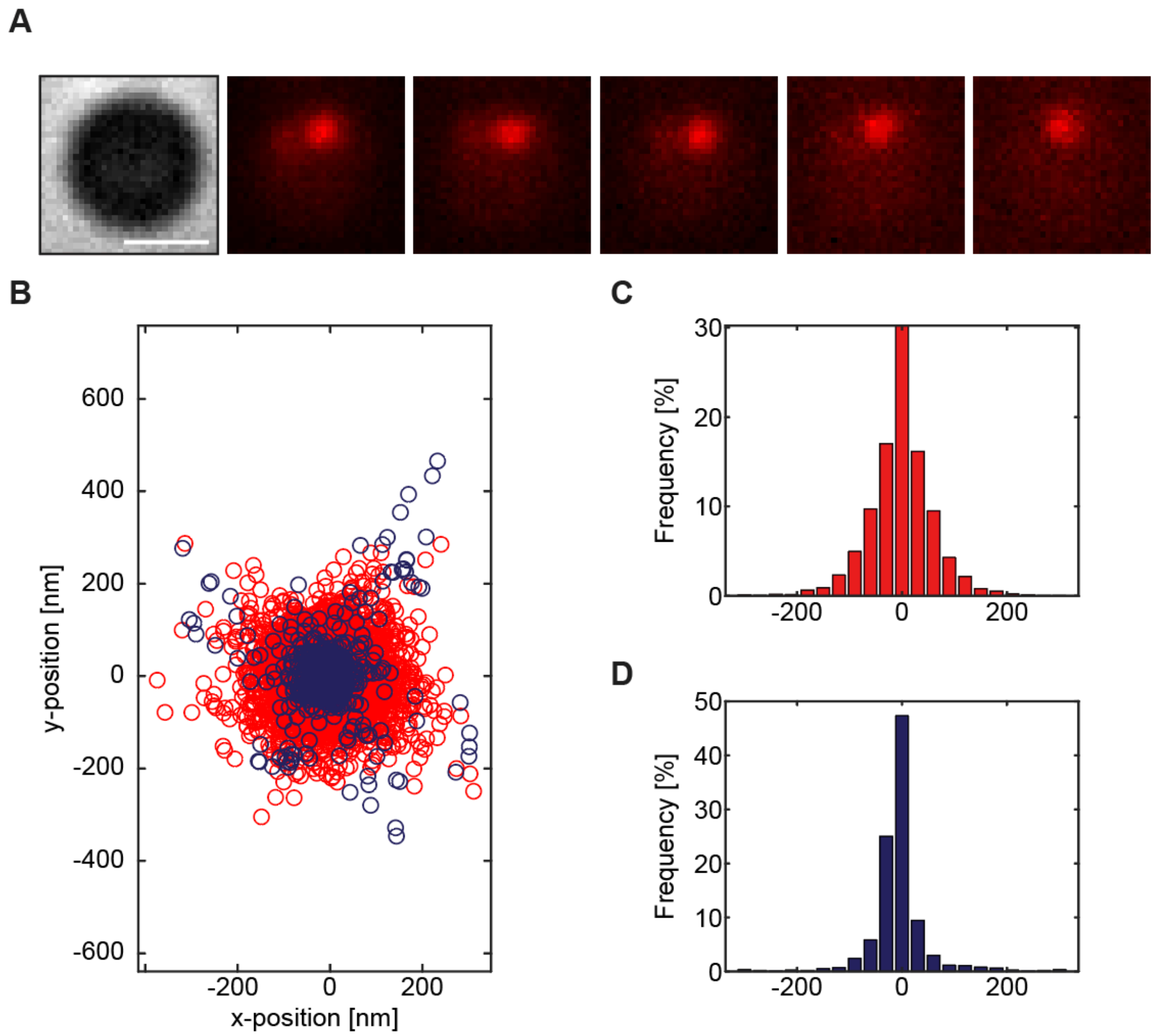

Figure S9. Dynamic movement of the origin of replication. A) Timelapse imaging of *ori* (ParB-mScarlet) movement within the expanded cell. Scale bar – 1 µm. Frame rate – 1 s. **B)** Tracked positions of ParB-mScarlet signal in all cells (red, N = 643) and marker cells (purple, N = 101). **C)** Distribution of the ParB-mScarlet signal relative to the initial position (x = 0 nm point) at frame zero. Full-width half maximum – FWHM = 89 nm. **D)** Same as in C) but for tracked cells (FWHM = 56 nm).

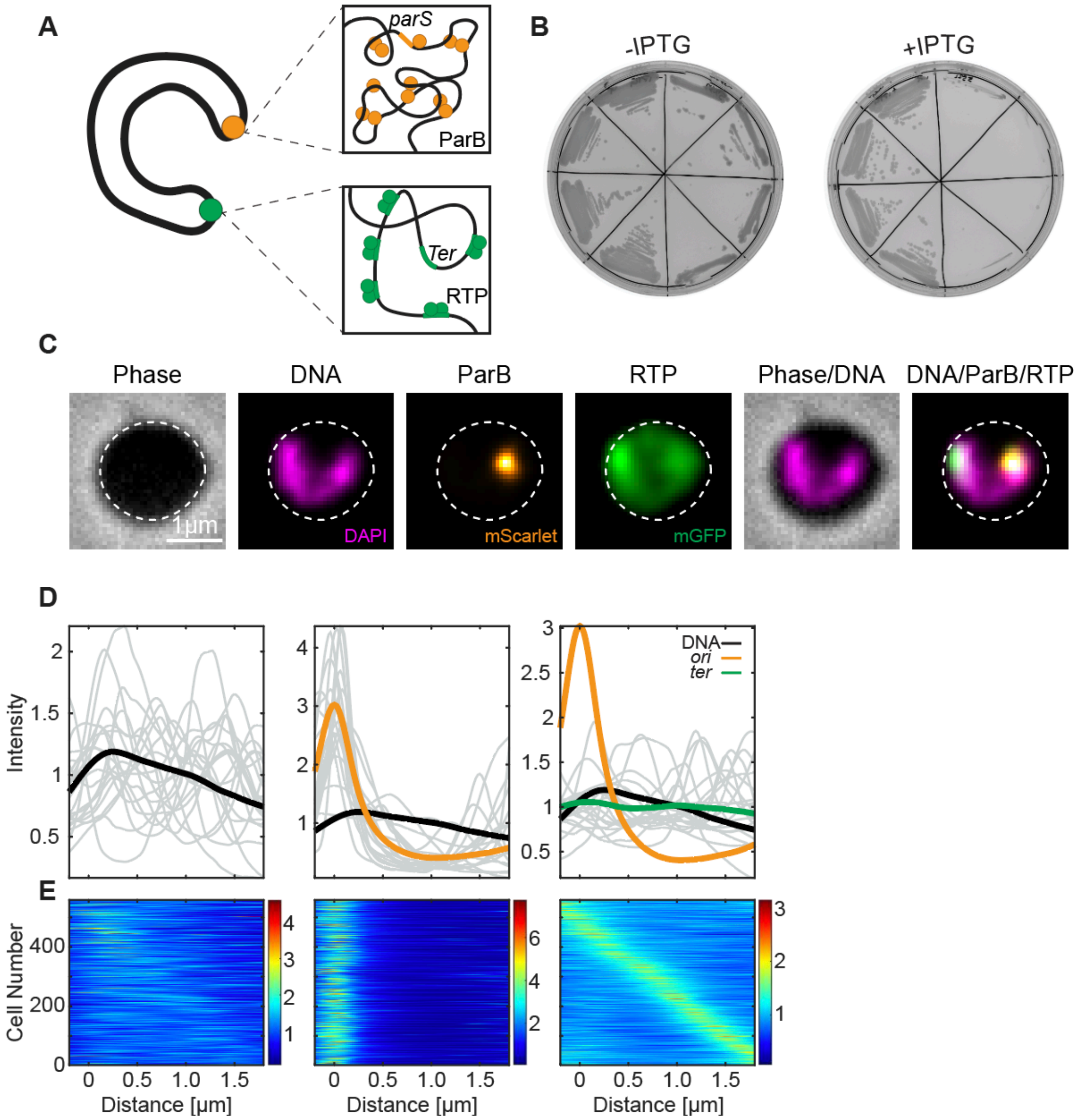

Figure S10. Origin and terminus of replication localize at different ends of the crescent chromosome. A) Graphical representation of the crescent-shape chromosome and the positions of two main chromosomal loci – *ori* and *ter*. **B)** Plating assay showing replication halt in the absence (left) or presence (right) of 2 mM IPTG for the strain BSG5522. **C)** Images of bacterial strain BSG5522 strain after lysozyme (400 μg/ml) treatment for 30 min. Scale bar = 1 μm. **D)** DNA intensity along the contour of the chromosome. Black, orange and green line shows the average normalized intensity obtained from all cells (N=559) for fluorescently labelled DNA, ParB and RTP signals, respectively. Gray lines display arbitrarily chosen individual examples. The position of the ParB focus is indicated on top (defining the 0 μm position). **E)** DNA density along the chromosome in all individual cells starting from the 0μm position which represents ParB/*ori*. Colorbar represents the fold-increase. Cells are ordered from top to bottom in terms of contrast.

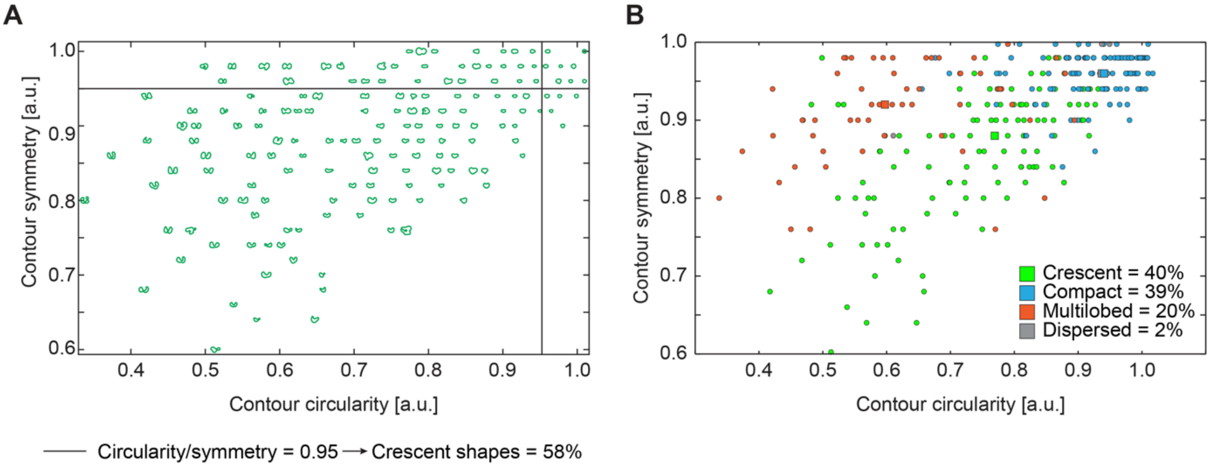

Figure S11. Quantification of crescent-shaped chromosomes in spherical cells after selection for single chromosomes. A) Cells plotted by their circularity and symmetry. Green line represents the Oufti object outline (which characterizes the chromosome contour). n = 292. Selection at threshold of 0.95 (full line) for the ration of circularity/symmetry results in 58% crescent shapes. **B)** Blinded manual classification of the chromosome shapes shown in panel A**.** The colored squares represent a mean value of symmetry and circularity estimates for the corresponding population. See Fig. S3 for additional context.

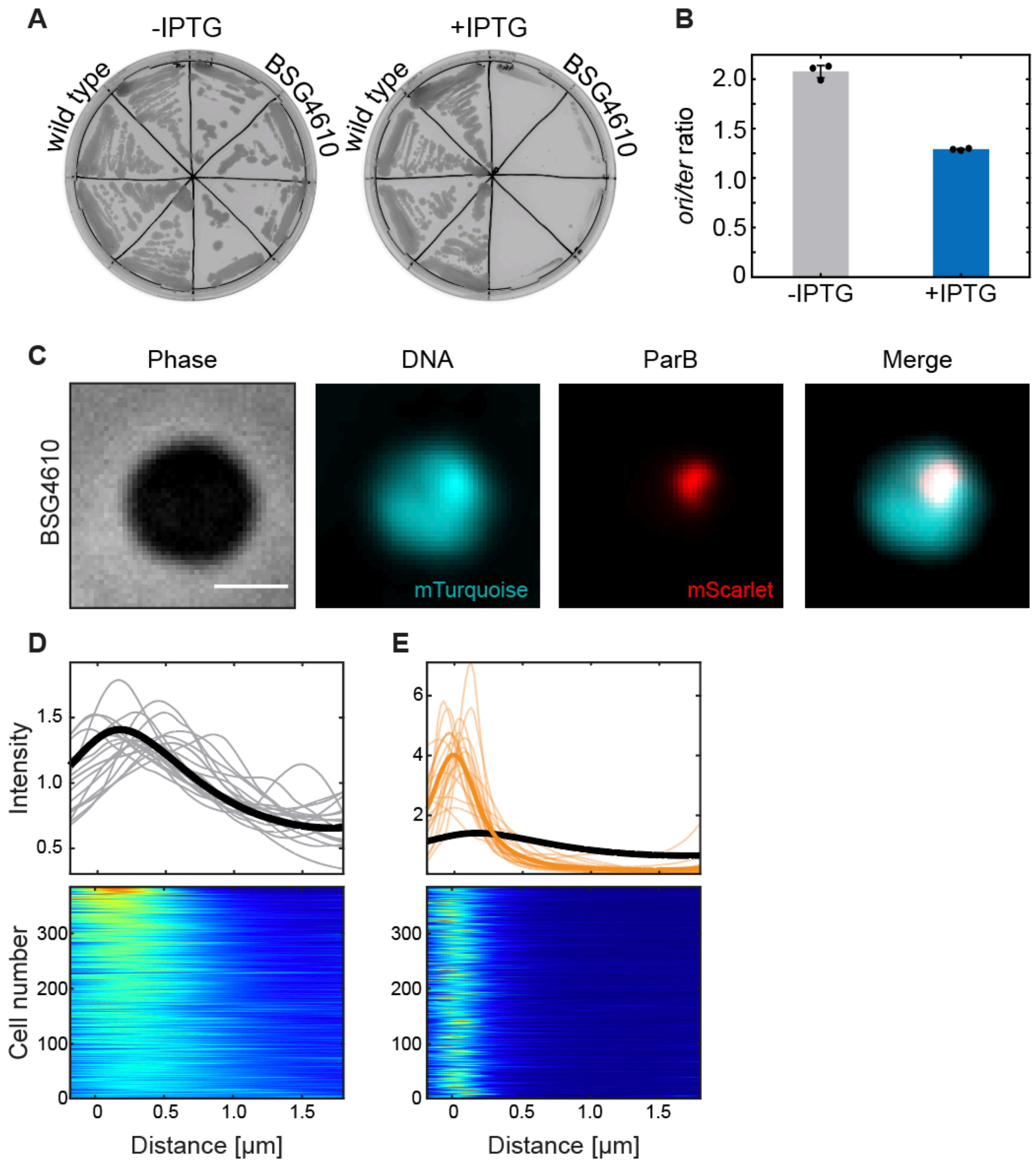

Figure S12. Origin of replication localizes within a region of high DNA density. A) Plating assay showing replication halt in the absence (left) or presence (right) of 2 mM IPTG. **B)** qPCR determination of *ori:ter* ratio in the strain BSG4610. Gray bar shows an untreated sample, and the blue bar shows a sample treated with 2 mM IPTG for 2.5 h. Both conditions were done in biological triplicates with technical triplicates for each of the colonies. Black dots represent and average value of a technical triplicate, for each biological triplicate. Error bars represent a standard deviation from three biological triplicates. **C)** Example of a BSG4610 cell after treatment with lysozyme (400 µg/ml) for 30 min. Scale bar = 1 µm. **D)** Top - DNA intensity along the contour of the chromosome. Black line shows the average normalized intensity obtained from all cells (N= 384). Gray lines display individual examples. The position of the ParB focus is set as the 0 µm position. Bottom - Corresponding density plots along the

chromosome in all individual cells. Results were pooled from biological duplicates. **E)** Same as in B for ParB-mScarlet signal. Thick orange line represents the averaged signal from all cells, while the thin orange lines represent arbitrarily chosen signals from individual cells.

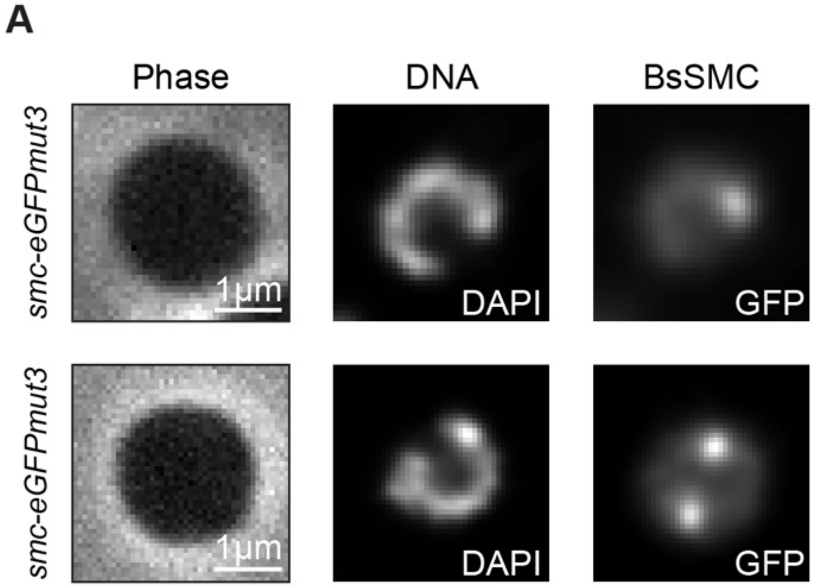

Figure S13. BsSMC proteins localize along the contour of the crescent shape and form fluorescent foci along it. A) Phase and fluorescence images of BSG4612 strain (see Table S1) containing P_{lac}-*sirA* and BsSMC-eGFPmut3 label. Top: example with a single BsSMC fowcus close to the tip of the crescent chromosome. Bottom: example with multiple BsSMC foci.

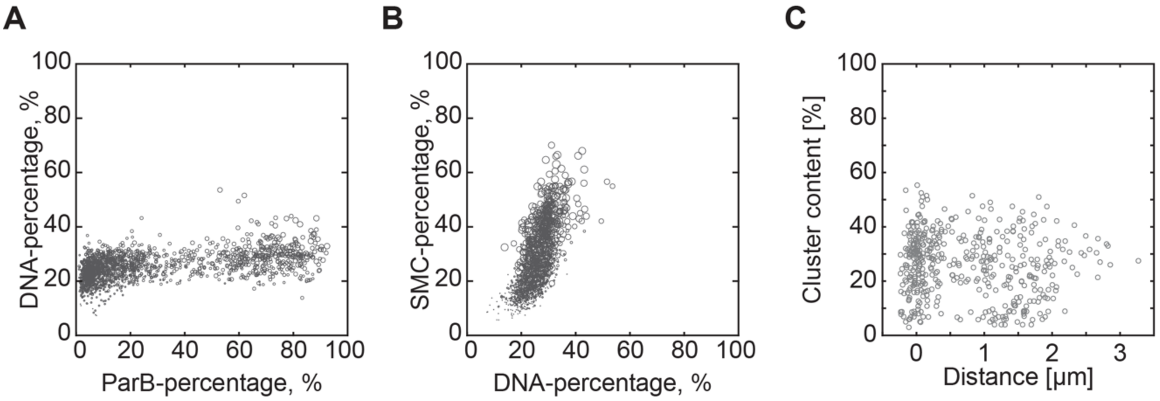

Figure S14. SMC proteins correlate with higher amount of DNA present. A) DNA amount within the primary cluster in relation to ParB intensity. **B)** SMC amount in relation DNA amount within the primary cluster. **C)** DNA content within secondary clusters of SMC proteins. Zero position represents the position of the secondary SMC cluster. Related to Fig. 3G.

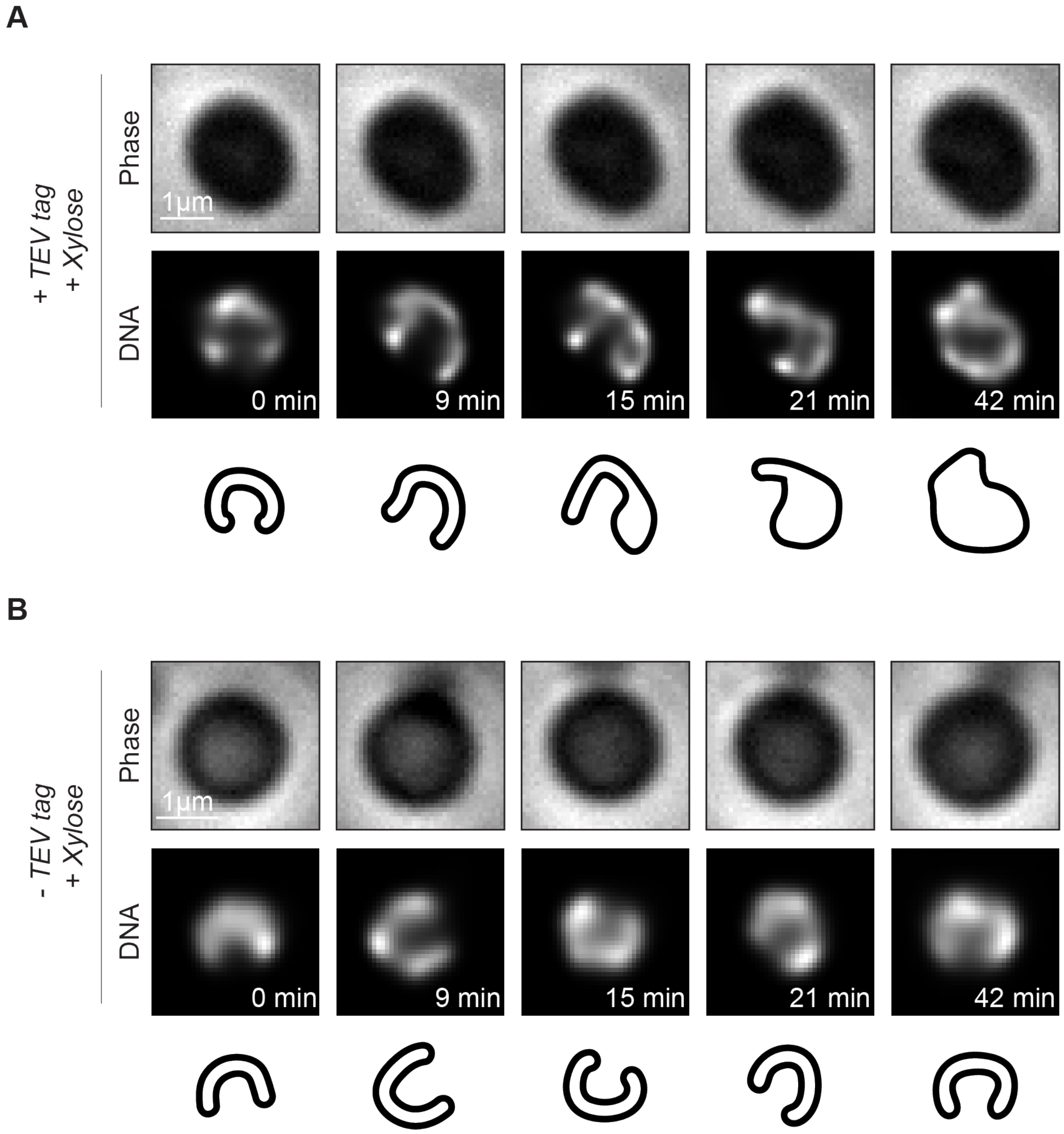

Figure S15. Real-time imaging of BsSMC protein knock-down via xylose-expressible TEV protease shows a reshaping of the chromosome which loses its crescent shape. A) Timelapse imaging ofa single *B. subtilis* **chromosome** in strain BSG219, containing the ScpA-TEV3 in presence of xylose-expressed wTEV protease (0.5% xylose). Schematic representation of chromosome shapes is represented below. **B)** Control experiment showing timelapse images under the same conditions as in A) but for strain BSG217 that does not contain the TEV tag and TEV protease.

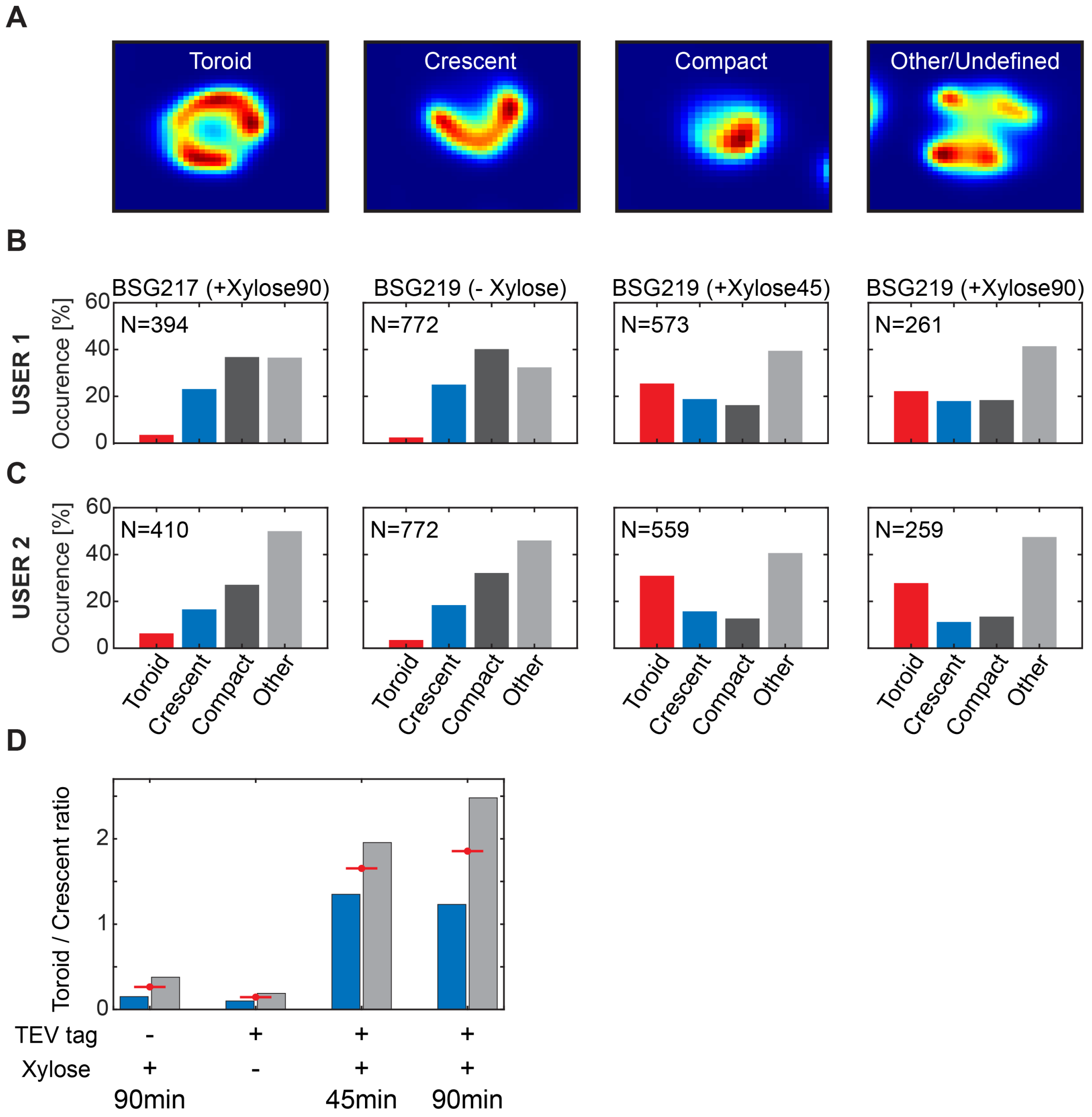

Figure S16. Identification of chromosome shapes under SMC knock-down conditions. A) Example images representing four chromosome categories that were presented to two independent users for blinded identification (see *Methods* for detailed description). **B)** Distribution of four selected categories over different samples (shown on top) for user 1 (MT). **C)** Same for user 2 (JK). **D)** Ratio of torus-shaped to crescent-shaped chromosomes in all samples. Blue and grey bars represent the two users; mean values of the two users are represented by the red lines. Related to Fig 4.

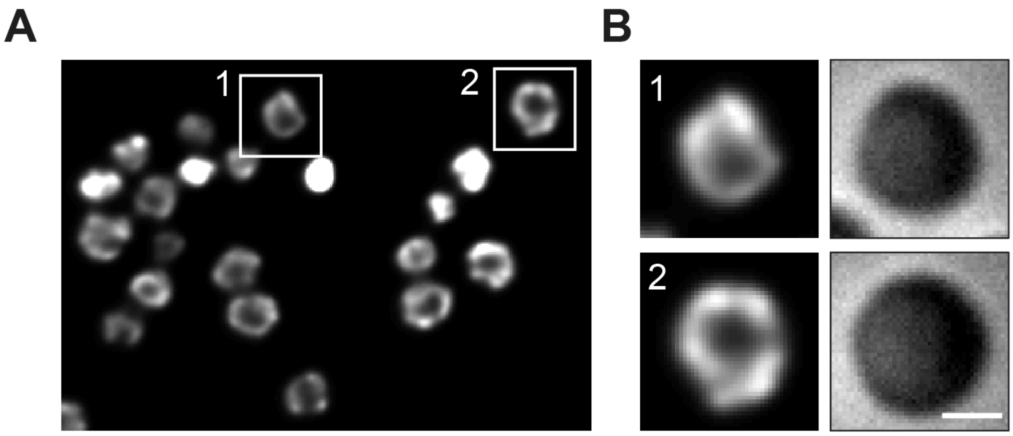

Figure S17. Chromosomes adopt a toroidal shape without chromosome arm-zipping in the absence of ParB protein. A) Representative image of BSG5503 (containing *ΔparB* mutation) strain after replication halt using IPTG (2 mM) for 2.5h. **B)** Zoomed regions from A) showing high-resolution images of toroidal chromosomes in the absence of *parB* locus. Scale bar = 1 µm.

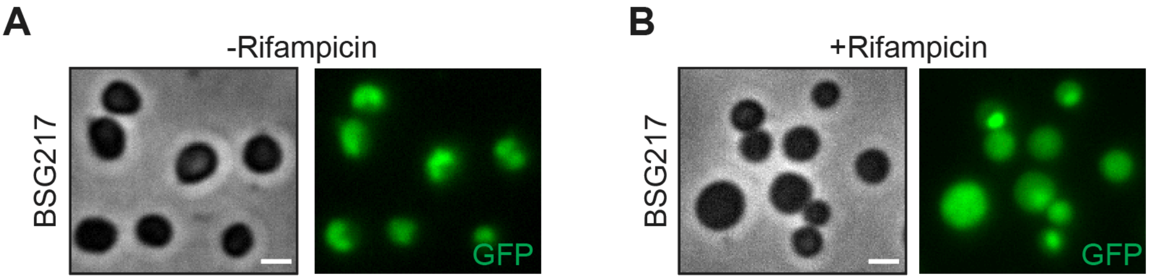

Figure S18. Transcription maintains the chromosome in a compact crescent shape. A) BSG217 (genotype) cells in the absence Rifampicin. **B)** Same cell line in the presence of 25µg/ml Rifampicin for 10 min. Scale bar = 2µm.
