# Supplementary material for: Direct observation of a crescent-shape chromosome in expanded Bacillus subtilis cells
Source: Nat Commun. 2024 Mar 28;15:2737. doi: 10.1038/s41467-024-47094-x (PMC10979009; doi:10.1038/s41467-024-47094-x)
Supplement: Supplementary file 3 — Description of additional supplementary files [file 41467_2024_47094_MOESM3_ESM.docx]

**Movie S1.**

**Title: *Bacillus subtilis* crescent-shaped chromosome in 3D-SIM microscopy*.***

**Movie S2.**

**Title: *Bacillus subtilis* crescent-shaped chromosome in 3D-SIM microscopy*.***

Description: Isosurface render of the 3D-SIM image, shown in Movie S1.

**Movie S3.**

**Title: Crescent chromosome is disrupted upon SMC knockdown*.***

Description: Chromosome image at time point t= 9 min. See Fig. 4 and Fig. S15 for more context.

**Movie S4.**

**Title: Crescent chromosome is disrupted upon SMC knockdown*.***

Description: Chromosome image at time point t= 42 min. See Fig. 4 and Fig. S15 for more context.
