# Supplementary material for: Direct observation of a crescent-shape chromosome in expanded Bacillus subtilis cells
Source: Nat Commun. 2024 Mar 28;15:2737. doi: 10.1038/s41467-024-47094-x (PMC10979009; doi:10.1038/s41467-024-47094-x)
Supplement: Supplementary file 8 — Reporting Summary [file 41467_2024_47094_MOESM8_ESM.pdf]

nature portfolio

Corresponding author(s): Cees Dekker

Last updated by author(s): Feb 29, 2024

Reporting Summary

Nature Portfolio wishes to improve the reproducibility of the work that we publish. This form provides structure for consistency and transparency in reporting. For further information on Nature Portfolio policies, see our Editorial Policies and the Editorial Policy Checklist.

Statistics

For all statistical analyses, confirm that the following items are present in the figure legend, table legend, main text, or Methods section.

n/a	Confirmed	
☐	☒	The exact sample size (*n*) for each experimental group/condition, given as a discrete number and unit of measurement
☐	☒	A statement on whether measurements were taken from distinct samples or whether the same sample was measured repeatedly
☐	☒	The statistical test(s) used AND whether they are one- or two-sided<br>*Only common tests should be described solely by name; describe more complex techniques in the Methods section.*
☒	☐	A description of all covariates tested
☒	☐	A description of any assumptions or corrections, such as tests of normality and adjustment for multiple comparisons
☐	☒	A full description of the statistical parameters including central tendency (e.g. means) or other basic estimates (e.g. regression coefficient) AND variation (e.g. standard deviation) or associated estimates of uncertainty (e.g. confidence intervals)
☐	☒	For null hypothesis testing, the test statistic (e.g. *F*, *t*, *r*) with confidence intervals, effect sizes, degrees of freedom and *P* value noted<br>*Give P values as exact values whenever suitable.*
☒	☐	For Bayesian analysis, information on the choice of priors and Markov chain Monte Carlo settings
☒	☐	For hierarchical and complex designs, identification of the appropriate level for tests and full reporting of outcomes
☐	☒	Estimates of effect sizes (e.g. Cohen's *d*, Pearson's *r*), indicating how they were calculated

Our web collection on statistics for biologists contains articles on many of the points above.

Software and code

Policy information about availability of computer code

Data collection

We carried out widefield Z-scans out using a Nikon Ti-E microscope with a 100X CFI Plan Apo Lambda Oil objective (NA=1.45). The imaging stage was maintained at 39°C for experiments using temperature-sensitive mutants, and at room temperature for mutants affected by IPTG expressing of SirA protein. We excited DAPI using SpectraX LED (Lumencor) filter cube with λex/ λbs/ λem = 363–391/425/435–438nm, GFP and SYTOXGreen using filter cube with λex/ λbs/ λem = 485-491 nm/506 nm/501-1100 nm, and mScarlet and STYOXOrange using filter cube with λex/ λbs/ λem = 540–580/585/592–668 nm. We used an Andor Zyla USB3.0 CMOS Camera to collect the fluorescent signals. We took 9-11 Z-slices of step size l = 200 nm, which accounts for a total of 1.8-2.2 µm of scanning volume.

For SIM microscopy, samples were prepared as previously described, except using two rounds of centrifugation with 3 ml cell culture in order to obtain higher cell density to accommodate for the smaller camera FOV. We used a Nikon Ti-E microscope with an AiRSIM module and a 100X CFI Apo Oil objective (NA=1.49). We used a 3D-SIM option with the 5 pos x 3 angles for imaging our live samples, and a Z-stack of 11 positions with l = 200 nm in between. NIS-Elements (version 5.2.1) software was used for image reconstruction where we carefully adhered to the Nikon N-SIM reconstruction guidelines and parameters to avoid reconstruction artifacts.

Data analysis

We used custom-written MATLAB scripts, modified from the original publication of Kaljevic et al., to extract the fluorescent foci positions, count the number of foci per cell, and determine the distances between foci in each channel. These scripts are open-source and available online (doi.org/10.5281/zenodo.7615509).

All fluorescent and phase images were visualized in the open-source image analysis software Fiji. We used a calculated microscopic pixel size of 65.35 nm/px in all images for scale bar creation. Adobe Illustrator 2020 was used for visualization and manuscript figures. After reconstructing the SIM microscopy images, we used SIMcheck software package for visualization and a Fiji in-built function “3D project” to create a 3D reconstruction of the crescent chromosome (Movie S1), as well as the plug-in 3D viewer to create the isosurface plots of the same image (Movie S2). The same was used to visualize the deconvolved 3D stacks in Movie S3-S4.

For deconvolution microscopy we used Huygens Professional deconvolution software (Scientific Volume Imaging, Hilversum, The

Netherlands), using an iterative Classic Maximum Likelihood Estimate (CMLE) algorithm. We measured the point spread function (PSF) experimentally by using 200 nm Tetraspeck beads (Invitrogen) and the recommended Huygens Professional guidelines (https://svi.nl/Point-Spread-Function-(PSF)). Stacks of 9-11 Z-slices were fed into the Huygens Professional software and we deconvolved each signal channel separately.

For manuscripts utilizing custom algorithms or software that are central to the research but not yet described in published literature, software must be made available to editors and reviewers. We strongly encourage code deposition in a community repository (e.g. GitHub). See the Nature Portfolio guidelines for submitting code & software for further information.

Data

Policy information about availability of data

All manuscripts must include a data availability statement. This statement should provide the following information, where applicable:

- Accession codes, unique identifiers, or web links for publicly available datasets
- A description of any restrictions on data availability
- For clinical datasets or third party data, please ensure that the statement adheres to our policy

All data included in the manuscript are available in the main text or the supplementary material. All raw microscopy data, raw data from plots, analysis scripts, including the test dataset for new users, will be freely available in Zenodo open repository (doi.org/10.5281/zenodo.7615509) upon publication

Research involving human participants, their data, or biological material

Policy information about studies with human participants or human data. See also policy information about sex, gender (identity/presentation), and sexual orientation and race, ethnicity and racism.

Reporting on sex and gender	n/a
Reporting on race, ethnicity, or other socially relevant groupings	n/a
Population characteristics	n/a
Recruitment	n/a
Ethics oversight	n/a

Note that full information on the approval of the study protocol must also be provided in the manuscript.

Field-specific reporting

Please select the one below that is the best fit for your research. If you are not sure, read the appropriate sections before making your selection.

☒ Life sciences ☐ Behavioural & social sciences ☐ Ecological, evolutionary & environmental sciences

For a reference copy of the document with all sections, see nature.com/documents/nr-reporting-summary-flat.pdf

Life sciences study design

All studies must disclose on these points even when the disclosure is negative.

Sample size	Sample sizes were determined by cell counting using image segmentation and analysis software Oufti (referenced in the main paper).<br><br>Sample sizes are always written within the Figure legend where used, as well as in Methods section. Exact number of cells used in a plot or analysis is mentioned in brackets in the figure legend of the corresponding plot as N=xx.
Data exclusions	No data was excluded from this work.
Replication	Experiments were performed using biological triplicates, if different, it is clearly stated in the figure legend.
Randomization	There is no randomization done in this study.
Blinding	We were unsuccessful in obtaining the ParB-mScarlet tag in strains BSG217 and BSG219, containing HbsU-tag, Pxyl-TEVp and/or ScpA-TEV3 in multiple cloning attempts. While we cannot distinguish between single and partially replicated chromosomes, due to strain construction issues, we attempted to quantitatively distinguish between torus-shaped chromosomes and crescent-shaped chromosomes in the presence of 0.5% xylose. For this, we performed the following manual user selection. We collected one image from four different samples (Fig. 4E, S16), which describe four experiments with different expected outcomes. Each of these was intensity thresholded to detect ROIs with separated DNA patterns based only on the continuous fluorescent signal. Next, a randomly picked ROI, containing a signal from a single cell, was presented to a user for classification of this pattern. The user could choose from one of four categories (toroid, crescent, compact, other/undefined) without knowing from which original image the ROI was taken. This was done for a series of 100 picks each, with no ROIs being presented to the user more than once within this series. To obtain an estimate for sampling variation, we repeated this series 20 times per user for a total of 2000 selected ROIs. To evaluate possible user bias, the procedure was done independently by two users (MT and JK) shown

in Fig. S16. We then traced back the described ROIs (in one of those four categories) to the original images and plotted the selection distribution shown in Fig. 4E and Fig. S16.

Reporting for specific materials, systems and methods

We require information from authors about some types of materials, experimental systems and methods used in many studies. Here, indicate whether each material, system or method listed is relevant to your study. If you are not sure if a list item applies to your research, read the appropriate section before selecting a response.

Materials & experimental systems

n/a	Involved in the study
☒	☐ Antibodies
☒	☐ Eukaryotic cell lines
☒	☐ Palaeontology and archaeology
☒	☐ Animals and other organisms
☒	☐ Clinical data
☒	☐ Dual use research of concern
☒	☐ Plants

Methods

n/a	Involved in the study
☒	☐ ChIP-seq
☒	☐ Flow cytometry
☒	☐ MRI-based neuroimaging

Plants

Seed stocks: n/a

Novel plant genotypes: n/a

Authentication: n/a
